# Dual omics comparison: how *Agrobacterium tumefaciens* and *Agrobacterium rhizogenes* modulate gene expression and metabolism in *Hypericum perforatum* L

**DOI:** 10.1186/s12864-025-12086-8

**Published:** 2025-10-24

**Authors:** Rajendran K. Selvakesavan, Maria Nuc, Matam Pradeep, Paweł Krajewski, Gregory Franklin

**Affiliations:** Institute of Plant Genetics of the Polish Academy of Sciences, Strzeszyńska 34, Poznań, 60-479 Poland

**Keywords:** *Hypericum perforatum*, *Agrobacterium* recalcitrance, Plant transformation, Plant defense, Gene transfer

## Abstract

**Background:**

*Agrobacterium* -mediated transformation is a fundamental method for the genetic modification of plants. However, several important crops and medicinal plants are recalcitrant to this process, hindering the application of modern functional genomics and genetic improvement tools. *Hypericum perforatum* L. (St. John’s wort), a valuable medicinal plant due to its secondary metabolites, is particularly recalcitrant to transformation mediated by *Agrobacterium tumefaciens*, and the molecular basis for this resistance remains unclear. This study was conducted to investigate the defense responses of *H. perforatum* after co-cultivation with *A. tumefaciens* and *Agrobacterium rhizogenes* through an integrative transcriptomic and metabolomic approach.

**Results:**

Transcriptome profiling revealed extensive reprogramming of gene expression in response to both *Agrobacterium* strains. Core genes for signal transduction, defense responses, transcriptional regulation and biosynthesis of secondary metabolites were strongly differentially expressed. In particular, WRKY, MYB and ERF transcription factor-encoding genes were induced, reflecting their role in triggering plant immunity. The upregulation of genes related to xanthone biosynthesis and the associated downregulation of flavonoid metabolism genes indicate a metabolic Shift towards xanthone production. Metabolomic analyses consistent with these results showed a striking increase in defense-related xanthones such as 6-deoxyisojacareubin, hyperxanthone E and gemixanthone A after treatment with *Agrobacterium*.

**Conclusions:**

*H. perforatum *possesses a controlled defense response to *Agrobacterium* that involves the transcriptional induction of defense signals and the accumulation of antimicrobial xanthones. The suppression of flavonoid biosynthesis also indicates a redirection of resources towards more efficient defense compounds. These results are important to elucidate the molecular basis of recalcitrance of *H. perforatum* transformation and to identify the role of pre-existing and inducible immunity in limiting *Agrobacterium*-mediated gene transfer.

**Supplementary Information:**

The online version contains supplementary material available at 10.1186/s12864-025-12086-8.

## Background

*Hypericum perforatum* L., commonly known as St. John’s wort, is a perennial herbaceous plant known for its therapeutic efficacy for various ailments, including depression. This is mainly due to its unique secondary metabolites such as hypericin, hyperforin, flavonoids and xanthones. These compounds confer potent pharmacological properties, including antidepressant, antimicrobial and anticancer effects, making *H. perforatum* a commercially and scientifically valuable medicinal plant [1, 2]. However, it is also known to be particularly resistant or recalcitrant to *Agrobacterium*-mediated genetic transformation [3]. The study by Franklin et al. (2008) also showed reduced viability of *Agrobacterium* after co-cultivation with *H. perforatum*. This resistance poses a major challenge for researchers trying to genetically improve this valuable species.

*Agrobacterium*-mediated transformation is an efficient and precise method of plant genetic engineering in which the desired gene is introduced into the plant genome *via* the natural ability of *Agrobacterium* species to transfer T-DNA from their Ti or Ri plasmids [4]. *Agrobacterium* also serves as a delivery tool for the components of clustered regularly interspaced short palindromic repeats (CRISPR)-associated systems, which enables efficient and targeted gene editing in plants [5]. However, some plant species, including *H. perforatum*, exhibit robust resistance to this process, often due to innate immune responses that limit bacterial colonization and DNA integration. Understanding the molecular basis of this resistance is crucial for the development of innovative transformation techniques or alternative approaches.

Plants have evolved highly coordinated defense systems to fight against various pathogens such as bacteria, fungi and viruses [6]. These defense responses include both constitutive and inducible mechanisms that act through pathogen recognition, signal transduction and changes in gene expression and metabolism [7]. The plant immune system functions on two main levels. The first, known as PAMP-triggered immunity (PTI), is triggered when pattern recognition receptors (PRRs) on the surface of plant cells recognize pathogen-associated molecular patterns (PAMPs) [8]. This triggers a cascade of defense signals including calcium influx, reactive oxygen species (ROS) production, activation of mitogen-activated protein kinases (MAPKs) and extensive transcriptional reprogramming [9]. The second level, known as effector-triggered immunity (ETI), involves intracellular resistance proteins that recognize specific pathogen effectors and often lead to stronger responses such as local cell death to limit the spread of the pathogen [8].

Recent developments in high-throughput “omics” technologies have significantly increased our knowledge of these immune processes. Transcriptomic studies show that plants respond to infection with large networks of genes related to hormone signaling (e.g. salicylic acid, jasmonic acid, ethylene), biosynthesis of secondary metabolites and production of defense-related proteins [10, 11]. For example, transcriptome analysis of cucumber plants infected with Cucumber Green Mottle Mosaic Virus showed strong suppression of RNA silencing pathways and activation of salicylic acid and ethylene signaling, indicating important shifts in the plant’s antiviral defense response [12]. Transcriptomic analysis of apples treated with the antagonistic yeast *Hannaella sinensis* revealed differential expression of key defense-related genes, particularly in hormone signaling, phenylpropanoid biosynthesis and plant-pathogen interaction pathways, indicating activation of host resistance mechanisms against pathogens [13]. A transcriptomic comparison between resistant and susceptible pepper cultivars revealed that resistance to *Xanthomonas campestris* pv. vesicatoria is associated with the specific induction of defense-related genes, including NBS-LRRs, WRKY and NAC transcription factors, and components of MAPK and calcium signaling pathways, revealing important molecular mechanisms underlying resistance to bacterial spot [14].

Metabolomics studies further contribute to the understanding of plant defense by elucidating dynamic changes in the composition and abundance of secondary metabolites that are crucial for immune responses [15]. Metabolome profiling in tomato revealed that inoculation with *Alternaria alternata* or chitin triggers a pattern-triggered chemical defense, characterized by the accumulation of specialized metabolites such as trigonelline, which has direct antifungal activity and may contribute to symptomless resistance [16]. Another metabolomic study on maize infected with *Setosphaeria turcica* showed extensive metabolic reprogramming, identifying over 1,200 different metabolites and a significant enrichment of metabolic pathways related to the biosynthesis of secondary metabolites and amino acids [17]. Metabolomic analyses of wheat infected with *Puccinia striiformis* f. sp. tritici revealed that homogentisic acid (HGA), a metabolite that is upregulated during infection, plays a functional role in disease resistance by inhibiting germination of fungal spores and reducing disease severity [18]. Metabolic profiling of *H. perforatum* cell suspensions treated with *Agrobacterium* elicitors revealed increased production of secondary metabolites, particularly phenolics, flavonoids and xanthones [19].

Metabolomic findings, when integrated with transcriptomic data, provide a comprehensive insight into the molecular and biochemical reprogramming underlying the plant defense mechanism. Integrated transcriptome and metabolome analyses of tobacco roots revealed that infection with *Ralstonia solanacearum* resulted in significant reprogramming of gene expression and metabolite profiles, with the resistant line showing upregulation of defense-related genes, particularly those in the phenylpropanoid pathway, and increased accumulation of coumarin-like metabolites and stress hormones, creating an overall less favorable environment for pathogen proliferation [20]. Combined transcriptome and metabolome analyses of poplar cultivars with different levels of resistance to *Lonsdalea populi* revealed that increased expression of genes related to photosynthesis and phenylpropanoid biosynthesis, together with timely accumulation of antibacterial metabolites such as catechin, contributed to increased resistance, while susceptible cultivars showed a trade-off between prolonged activation of defense and suppression of growth-related processes [21].

This study combines these two “omics” approaches to unravel the complex defense mechanisms of *H. perforatum* against *Agrobacterium* infection. In our study, cell suspension cultures of *H. perforatum* were used to allow high reproducibility and uniform exposure to *Agrobacterium*. Similar approaches have been applied in model systems such as the BY-2 cell Line of tobacco, where nearly 100% of the cells could be synchronously infected and analyzed for early transcriptomic changes after *Agrobacterium* exposure [22]. By analyzing the transcriptional and metabolic responses at different time points after treatment, the study reveals the molecular events underlying the recalcitrance of this medicinal plant to genetic transformation. Ultimately, this work contributes to a better understanding of plant immunity and provides strategic insights to overcome the challenges of *Agrobacterium*- mediated transformation in recalcitrant species.

## Methods

### Establishment of the cell suspension culture

Cell suspension cultures of *H. perforatum* obtained from the cultivar ‘Helos’ (Richters seeds, Ontario, Canada) were used for the present study. The suspension cells were maintained in Erlenmeyer flasks (250 mL) containing Murashige and Skoog (MS) medium (Duchefa Biochemie, Haarlem, The Netherlands) supplemented with 0.5 mg/L naphthalene acetic acid. The cells were grown on an orbital shaker (Benchmark Scientific, USA) at 110 rpm in a growth chamber with a photoperiod of 16/8 (day/night), an irradiation of 80 µmol m^−^²s^−^¹, relative humidity of 70% and a temperature of 25°C. To keep the cells in the growth phase, 10 mL of the grown culture was subcultured into 70 mL of fresh medium every 7 days.

### Agrobacterium culture

*Agrobacterium tumefaciens* strain EHA105 and *Agrobacterium rhizogenes* strain LBA1334, each carrying the plasmid pCAMBIA2301, were grown separately in Luria-Bertani (LB) medium (L3022, Sigma-Aldrich) supplemented with 50 mg/L kanamycin (K0126, Duchefa Biochemie) and 20 mg/L rifampicin (R0146, Duchefa Biochemie). A single bacterial colony was used to inoculate 5 mL of broth, which was incubated for 48 hours (h) at 28 °C with shaking at 180 rpm (KS 3000 ic control, IKA). This starter culture was then expanded to 200 mL. Once the optical density (OD) of the culture reached 0.6–0.8 at 600 nm, it was centrifuged at 1240 × *g* (Eppendorf Centrifuge 5804) to collect the bacterial cells. The supernatant was discarded and the bacterial pellet was resuspended in MS medium to prepare a bacterial stock solution.

### Co-cultivation of bacteria and plant cells

*A. tumefaciens* was added to *H. perforatum* cell suspension cultures in the exponential phase (three to five days after subculture) to achieve a final OD of 1.0 at 600 nm. Three biological replicates for the treatment and control were incubated in a growth chamber with a photoperiod of 16 h of Light and 8 h of darkness at 25 °C, an irradiation of 80 µmol m^−^²s^−^¹ and a relative humidity of 70%, and both the treated and control cultures were kept alive. After co-cultivation of *H. perforatum* and *A. tumefaciens*, the cells were collected at different time points (0.5 h, 3 h, 12 h, 24 h). After the cells were vacuum filtered from the medium, they were immediately cryopreserved in liquid nitrogen and stored at −80 °C. The same procedure was used for the co-cultivation of *H. perforatum* and *A. rhizogenes*.

### RNA extraction and sequencing

The freshly harvested biomass from the cell suspension culture and plantlets was ground to a fine powder using Liquid nitrogen in a sterile mortar and pestle. Total RNA was isolated from 100 mg of biomass using the Sigma Spectrum RNA extraction kit (Sigma-Aldrich, USA). DNase treatment (Sigma-Aldrich, USA) was used to remove all trace DNA according to the manufacturer’s recommendations. The Agilent Bioanalyzer 2100 was used to check RNA integrity, while the NanoDropTM OneC was used to measure the amount of RNA. High quality RNA was extracted from the control and treated cell suspension cultures of *H. perforatum* and RNA samples with an RNA integrity number (RIN) of at least seven were used for RNA-seq analysis. Random hexamer primers were used to synthesize the cDNA after random fragmentation of the mRNA. DNA polymerase I, dNTPs, RNase H and a special second-strand synthesis buffer (Illumina) were used to generate the second strand. After size selection, the double-stranded cDNA was purified and 150 bp paired-read Illumina sequencing was performed (Novogene, Beijing, China).

### Data analysis

The RNA-seq data were used to quantify the expression of 88 616 transcripts from the reference library described in [23] using Salmon v. 0.12.032 [24] in mapping mode. Removal of sequences Linked to adapters and quality trimming were performed using AdapterRemoval ver 2.1.7 [25]. The resulting count data were subjected to differential expression analysis in DESeq2 ver. 1.22.2 [26]. Differentially expressed transcripts between treated and control samples were identified as those that had a mean expression of at least 20 units (estimated in DESeq2), log2(Fold Change) > 2 and FDR < 0.01 (Benjamini-Hochberg adjusted P- values). Transcripts were annotated using OmicsBox version 2.0.10 (https://www.biobam.com/omicsbox), with BLAST searches performed against the non-redundant (nr) protein database restricted to the taxon Magnoliopsida (e-value threshold of 1e-10). Additional functional analyses, including the assignment of Gene Ontology (GO) terms, were performed using the tools available in the functional analysis suite of the software. Volcano plots and Gene Ontology graphs were constructed using SR Plot [27].

### Metabolomic analysis

#### Sample preparation and extraction

The cryopreserved *H. perforatum* cells were ground to a fine powder under pre-chilled conditions and subsequently lyophilized in a freeze dryer (Heto-Holten A/S, Denmark). For extraction, 100 mg of the dry powder was mixed separately with 1 mL of 80% methanol in amber Eppendorf tubes to protect the Light-sensitive compounds. The mixtures were vortexed at 5000 rpm for 10 minutes (min) and then subjected to ultrasonic extraction in Bandelin Sonorex bath for 10 min at room temperature. Following sonication, samples were centrifuged for 20 min at 20 °C and 25,150× *g* in a Hettich EBA 21 centrifuge equipped with 1024 rotor. The resulting supernatant was filtered through 0.45 μm discs of PTFE syringe filters (13 mm, Kinesis, Vernon Hills, USA) into glass vials and stored at −80 °C for further analysis.

#### Identification and quantification of the compounds

Compounds were identified by analyzing 1 µL of each extract using a Waters Acquity UPLC system in combination with a Q-Exactive Orbitrap mass spectrometer (Thermo Scientific, Bremen, Germany). Chromatographic separation was carried out using a binary solvent system consisting of mobile phase A (0.1% formic acid in water) and mobile phase B (acetonitrile: methanol, 80: 20, v/v). The separation was performed on a UPLC-Cortecs BEH Shield C18 column (100 × 2.1 mm, particle size 1.7 μm) at 35 °C and a flow rate of 400 µL/min. The autosampler was set to 5 °C. The elution gradient was programmed as follows: Starting at 2% B, Linear increase to 80% B over 11 min, followed by an isocratic hold at 99.5% from 12 to 16 min, returning to 2% B after 16.5 min for re-equilibration. Data acquisition and system control were performed using TUNE 2.8 and XCalibur 4.0 software (Thermo Scientific, Bremen, Germany). Mass spectrometric detection was conducted in negative mode, with full-MS scans recorded over an *m/z* range of 100–1500 at a resolution of 70,000. Compounds were identified based on the exact *m/z* value, retention time and MS/MS fragmentation patterns as described in a previous study [28]. Quantification of compounds in the extracts was performed by UPLC in conjunction with photodiode array (PDA) detection. A 3 µL aliquot of each extract was injected for analysis. Detection was performed using 2D chromatography channels at a fixed wavelength of 270 nm. The retention times obtained from LC-MS analysis were used to identify the corresponding peaks in the chromatograms. The chromatographic data, including retention times and peak areas were processed and exported to Excel spreadsheets for quantification. An external standard method was used to quantify target compounds. In the absence of authentic standards, structurally similar compounds were used as reference standards: Dihydroxybenzoic acid for benzoates and benzophenones, caffeic acid for hydroxycinnamic acid (HCA) derivatives, kaempferol for flavonoids, mangiferin for xanthones, prenylated xanthones and xanthonoids.

### Statistics

For the statistical analysis, the concentrations of all quantified compounds from the respective extracts were grouped accordingly. Three biological replicates (*n* = 3) were used to calculate the mean and standard error of the mean (SEM). To detect significant differences in the concentration of secondary metabolites between the control and treated samples, a two-way ANOVA followed by Dunnett’s test for multiple comparisons was performed using Graphpad Prism 10 software. A significance level of *P* < 0.05 was considered statistically significant.

## Results

### Transcriptomic data analysis

Good quality raw RNA-seq data were obtained for 35 out of 36 planned samples (3 treatments × 4 time points × 3 replicates), with the number of reads ranging from 40.2 to 82.1 million and the proportion of mapped reads ranging from 61.4 to 74.7% (Table S1); such mapping proportions are considered satisfactory when the (*de novo*) transcriptome is used as a reference [29]. No outlying samples were identified (Fig. S1). Pearson correlation coefficients between biological replicates, calculated using count data for all reference transcripts, were low for one out of three samples within five experimental variants (not clustered inside any level of Treatment or Time) (Fig. S2). These samples were not removed from the analysis to ensure that the data used for the differential gene expression analysis represented true biological variability. The analysis was done also for the reduced dataset obtained by excluding the five low-correlated replicates; the differences between results obtained in two analyses were not substantial (Fig. S3), which suggests that the data quality was sufficient for the inference.

### Differential expression of H. perforatum transcripts in response to Agrobacterium treatment

**Fig. 1:**
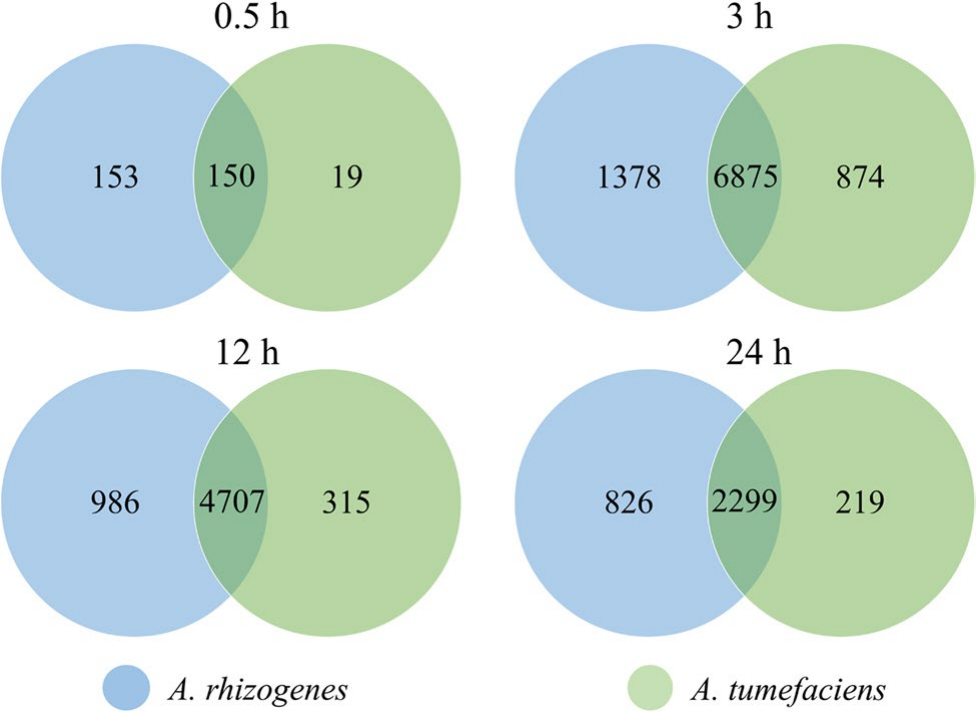
Venn diagram showing the number of differentially expressed transcripts in *H. perforatum* cells after treatment with *A. tumefaciens* and *A. rhizogenes* at different time points

RNA-seq analysis of *H. perforatum* cells exposed to *Agrobacterium* species (*A. tumefaciens* and *A. rhizogenes*) revealed extensive and significant changes in gene expression across time points (Table S2). While each bacterial species induced different changes in gene expression, substantial overlap was observed in differentially expressed transcripts was observed, suggesting activation of common defense and signaling pathways (Table 1; Fig. 1).

**Table 1:** Differential expression of *H. perforatum* transcripts after *Agrobacterium* treatment

Bacteria	Expression	Number of genes differentially expressed			
		0.5 h	3 h	12 h	24 h
*A. rhizogenes*	**Upregulated**	292	4056	2329	1574
	**Downregulated**	11	4197	3364	1551
*A. tumefaciens*	**Upregulated**	164	3894	2111	1261
	**Downregulated**	5	3855	2911	1257

**Fig. 2:**
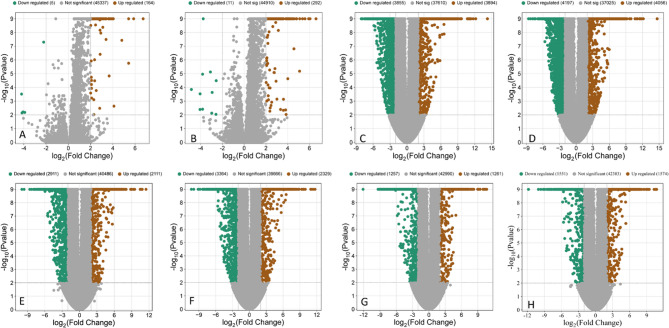
The volcano plots showing upregulated and downregulated genes. **A** *A. tumefaciens*, 0.5 h; (**B**) *A. rhizogenes*, 0.5 h; (**C**) *A. tumefaciens*, 3 h; (**D**) *A. rhizogenes*, 3 h; (**E**) *A. tumefaciens*, 12 h; (**F**) *A. rhizogenes*, 12 h; (**G**) *A. tumefaciens*, 24 h; (**H**) *A. rhizogenes*, 24 h

The volcano plots show a dynamic transcriptional landscape across four time points in *H. perforatum* after *Agrobacterium* treatment. At the earliest time point (0.5 h), responses were modest, with 292 upregulated and 11 downregulated genes in *A. rhizogenes*-treated cells, compared with 164 upregulated and 5 downregulated genes in *A. tumefaciens*-treated cells (Fig. 2), reflecting initial recognition of the pathogen accompanied by limited upregulation of pathogenesis-related (PR) genes and receptor transcripts. Transcriptional activity peaked at 3 h, with a striking 6,875 genes showing differential expression when treated with both bacterial species (Fig. 2 and Table S2). After 12 and 24 h, the differential gene expression decreased, indicating a stabilization phase characterized by sustained production of secondary metabolites and gradual down-regulation of signaling pathways. Overall, these observations emphasize the temporal coordination of metabolic and immune responses in *H. perforatum*.

**Fig. 3:**
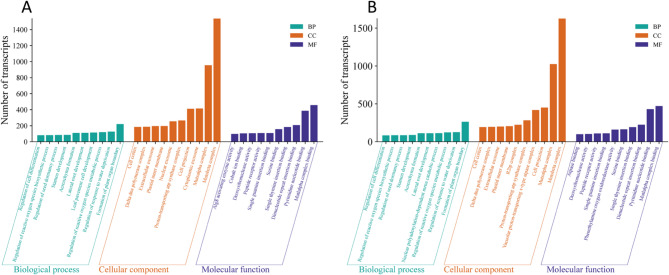
Histograms of GO function analysis for differentially expressed genes after 3 h of *Agrobacterium* treatment (**A**. *A rhizogenes*; **B** *A. tumefaciens*); the top ten GO terms with the highest number of transcripts were shown for each classification

We performed Gene Ontology (GO) enrichment analysis for both *A. rhizogenes* and *A. tumefaciens* to investigate the functional landscape of differentially expressed genes. At the 3 h time point when the highest number of differentially expressed genes was observed, a total of 1,388 biological processes (BP), 314 cellular components (CC), and 1,108 molecular functions (MF) were enriched in the *A. rhizogenes* treatment, while the *A. tumefaciens* treatment yielded 1,332 BP, 282 CC, and 1,051 MF terms. The top 10 GO terms from each category are shown in Fig. 3. Enriched biological processes were associated with important plant defense strategies, including regulation of metabolic processes of reactive oxygen species, metabolic processes, biosynthetic processes of reactive oxygen species, regulation of response to water deprivation and lateral roots, indicating oxidative stress signaling. In the category of cellular components, terms such as the inner plastid membrane, the vacuolar proton-transporting ATPase complex and extracellular exosomes indicate localized defense activities in energy and transport systems. Molecular functions such as peptide receptor activity and deoxyribonuclease activity also suggest a role in signal perception and nucleic acid metabolism. GO analyses for the other time points (0.5 h, 12 h and 24 h) can be found as supplementary figures (Fig. S4).

**Fig. 4:**
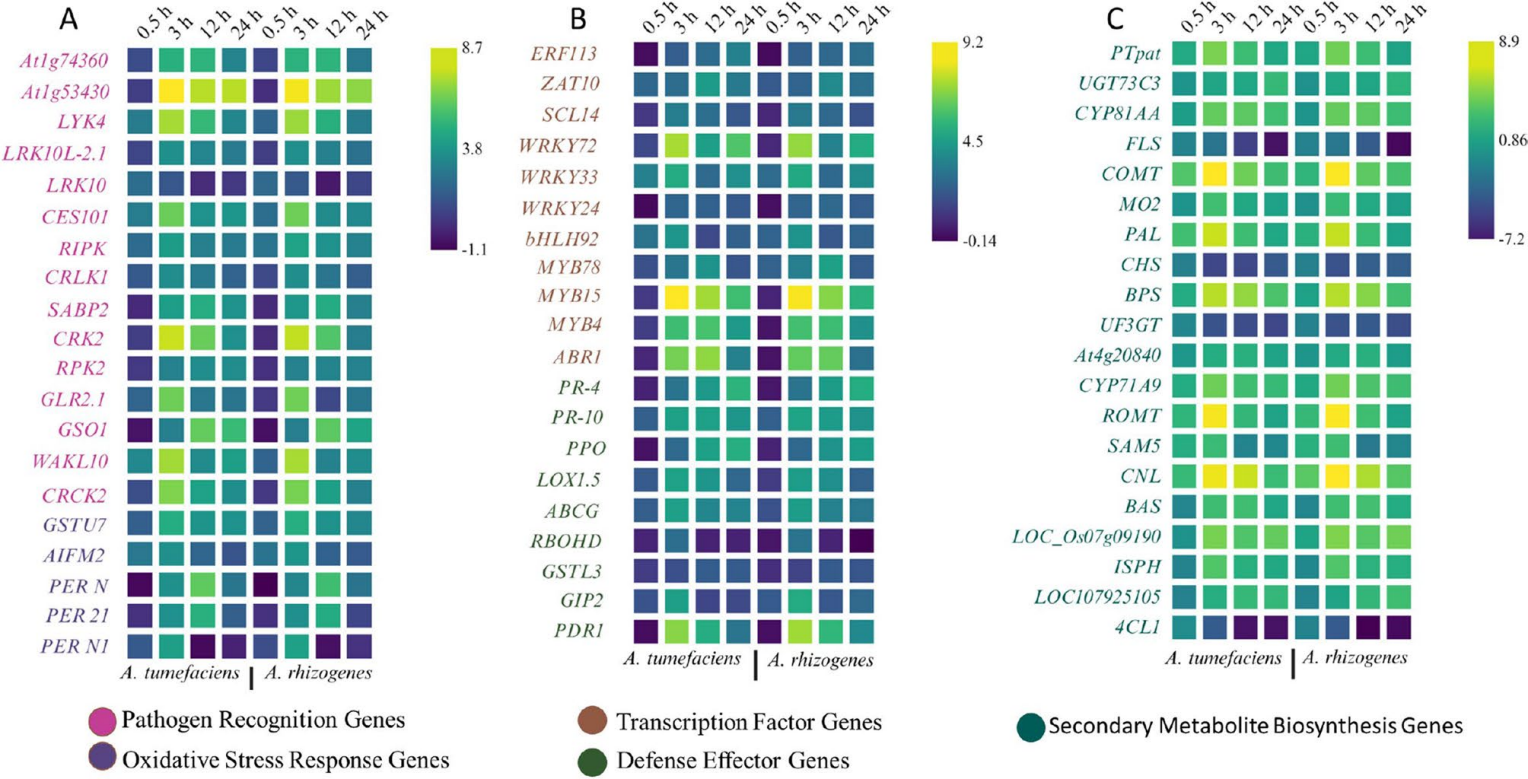
Heatmap of the most differentially expressed transcripts. **A** Transcripts of pathogen recognition genes, signal transduction genes and oxidative stress response genes; (**B**) Transcripts of transcription factors and defense effectors; (**C**) Transcripts of genes for secondary metabolite biosynthesis. The colour scale represents the average log2(fold change) of gene expression. Full names corresponding to gene symbols used in the heat map are provided in Table S3

*Agrobacterium* treatment resulted in differential expression of genes which we broadly categorized as pathogen recognition, signal transduction, oxidative stress response, transcription factors, defense effectors, and biosynthesis of secondary metabolites based on the transcriptomics results (Fig. 4).

### Metabolomic analysis

**Fig. 5:**
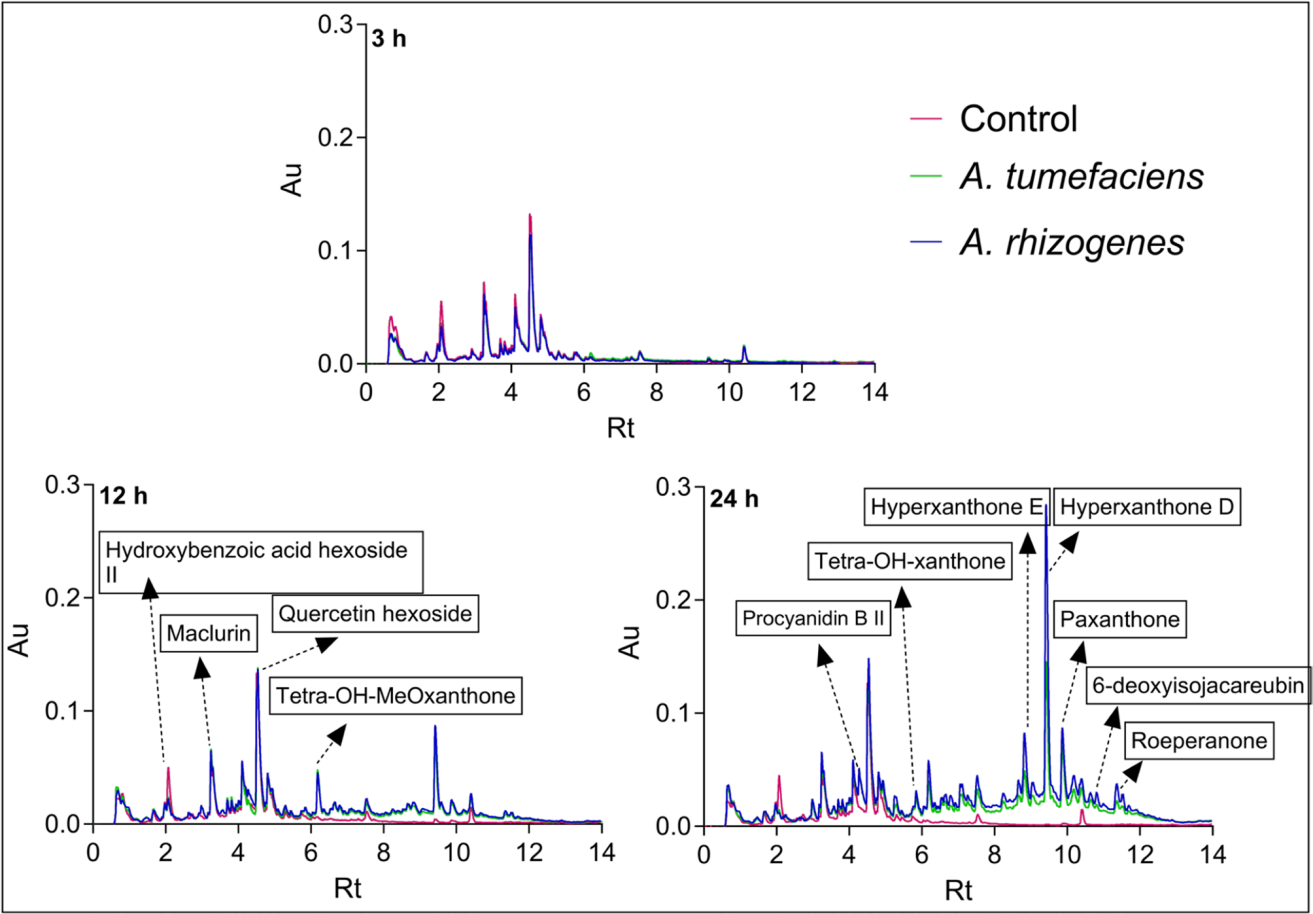
UPLC-PDA chromatograms recorded at 270 nm showing the effects of *A. tumefaciens* and *A. rhizogenes* treatments on the secondary metabolite profiles of *H. perforatum* cells

Metabolome profiling using UPLC-PDA and high-resolution mass spectrometry revealed 48 differentially accumulated metabolites (Fig. 5; Table S4) after co-cultivation, with 14 compounds (Table 2) clearly induced after *Agrobacterium* treatment. These compounds belonged predominantly to the class of xanthones, which are known for their antioxidant and antimicrobial properties. Among the metabolites, procyanidin B II showed the most striking increase after 24 h, together with other bioactive xanthones such as hypericophenonoside I, hyperxanthone E and tetra-hydroxy-xanthone. These compounds were not present in untreated samples and showed a clear accumulation in treated cells.

**Table 2:** Antioxidant/antimicrobial compounds produced in *H. perforatum* cells in response to *A. tumefaciens* and *A. rhizogenes* (C- control, AT- *A. tumefaciens* and AR- *A. rhizogenes*)

	Compound	Concentration (µg/g DW)								
Rt		3 h			12 h			24 h		
		C	AT	AR	C	AT	AR	C	AT	AR
2.25	Hypericophenonoside I	0	0	0	0	0	0	0	120 ± 11^*^	140 ± 8.5^*^
4.28	Procyanidin B II	0	0	0	0	80 ± 16^*^	300 ± 14^*^	0	433 ± 18^*^	494 ± 16^*^
4.96	TetraOH, MeOxanthone hexoside	0	0	0	0	44 ± 8.7^*^	61 ± 1.5^*^	0	80 ± 3.2^*^	100 ± 2.8^*^
5.84	Tetra-OH-xanthone	0	0	0	0	48 ± 14^*^	56 ± 2.7^*^	0	139 ± 6.8^*^	173 ± 6.1^*^
6.66	Bisxanthone	0	0	0	0	43 ± 9.2^*^	59 ± 2.1^*^	0	66 ± 2.8^*^	82.1 ± 4.4^*^
6.78	Hyperxanthone B	0	9.3 ± 1^*^	7.4 ± 1.2^*^	0	45 ± 9.9^*^	72 ± 3^*^	0	56 ± 1.9^*^	93.8 ± 19^*^
8.06	Hyperxanthone C	0	0	0	0	27 ± 7.2^*^	47 ± 7.5^*^	0	74 ± 6.6^*^	87.6 ± 3.3^*^
8.24	DiHO, diMeOxanthone 4	0	0	0	0	29 ± 7.8^*^	45 ± 2.2^*^	0	87 ± 1.8^*^	117 ± 6.7^*^
8.41	Gemixanthone A	0	0	0	0	23 ± 6^*^	39 ± 1.8^*^	0	56 ± 2.5^*^	74.4 ± 5.1^*^
8.65	Toxyloxanthone B	0	0	0	0	31 ± 7.7^*^	58 ± 2.1^*^	0	127 ± 4.1^*^	165 ± 10^*^
8.82	Hyperxanthone E	0	2.7 ± 0.5^*^	2.7 ± 1^*^	0	57 ± 14^*^	91 ± 3.8^*^	0	192 ± 6.9^*^	259 ± 26^*^
10.89	6-deoxyisojacareubin	0	0	0	0	0	0	0	65 ± 3.5^*^	91.1 ± 5.9^*^
10.98	DiHO, diMeOprenylxanthone	0	0	0	0	0	0	0	69 ± 3.1^*^	96.4 ± 5.6^*^
11.91	Roeperanone	0	0	0	0	0	0	0	75 ± 6.4^*^	109 ± 6.4^*^

All values given are mean ± SEM of three biological replicates. Dunnett’s multiple comparisons show a significant difference in means at *P* < 0.05 (*)

### Integrated transcriptome–metabolome analysis

To further elucidate the relationship between transcriptomic changes and secondary metabolite accumulation in *H. perforatum* upon *Agrobacterium* infection, we conducted an integrated analysis of gene expression and metabolite abundance, focusing on flavonoid and xanthone biosynthesis pathways. We quantified the relative carbon flow through the two competing branches of phenylpropanoid metabolism, flavonoid and xanthone biosynthesis by normalizing the sum of all measured flavonoids and xanthones to 100% in each treatment condition.

**Fig. 6:**
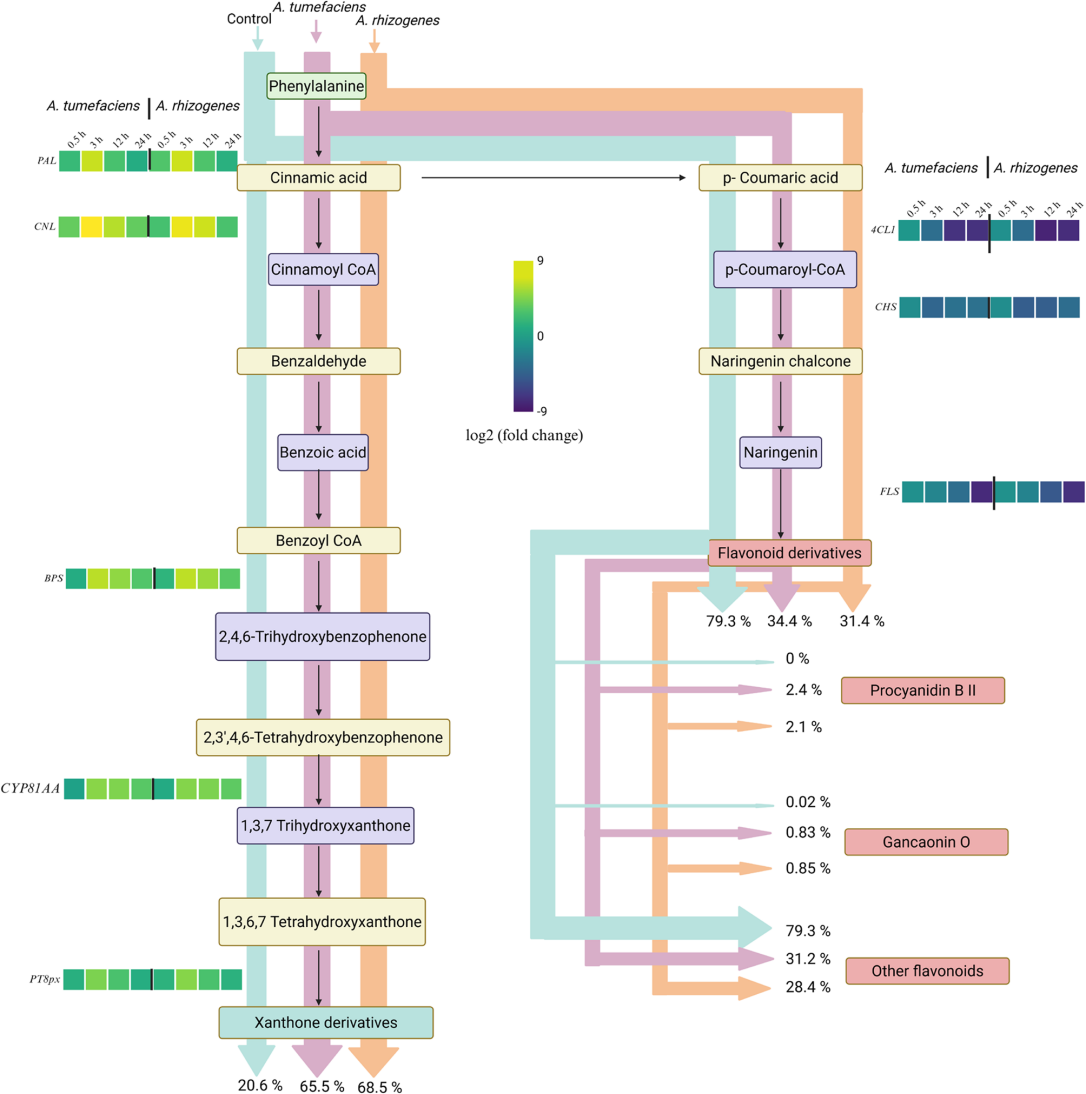
Reprogramming of flavonoid and xanthone biosynthesis in *H. perforatum* under *Agrobacterium* stress. The sum of total flavonoids and xanthones was normalized to 100% for each condition to represent the relative proportion of each metabolite. Heatmap represents the gene expression for each metabolic enzyme according to the indicated colour scale

Our analysis revealed a striking reallocation of metabolic flux in response to *Agrobacterium* stress (Fig. 6). In control tissues, 79.3% of carbon flow was directed toward non-specific flavonoid derivatives, with only 20.6% directed toward xanthone biosynthesis. However, *A. tumefaciens* and *A. rhizogenes* treatments resulted in a dramatic increase in the proportion of xanthone derivatives, accounting for 65.5% and 68.5%, respectively. Concurrently, the share of flavonoid derivatives declined to 34.4% (*A. tumefaciens*) and 31.4% (*A. rhizogenes*), indicating a metabolic shift favoring xanthone production.

Importantly, while most flavonoids were downregulated under *Agrobacterium* treatment, two specific antimicrobial flavonoid compounds including procyanidin B II (2.4% and 2.1%) and gancaonin O (0.83% and 0.85%) were induced. This suggests a selective enhancement of flavonoids with potential defensive properties, while the broader flavonoid biosynthetic pathway is suppressed.

The metabolic shift is supported by transcriptomic evidence. Genes associated with xanthone biosynthesis, such as *phenylalanine ammonia lyase* (*PAL*), *benzoyl-CoA 2-hydroxylase* (*BPS*), *1*,*3*,*7-trihydroxyxanthone synthase*, and *8-prenyl-1*,*3*,*6*,*7-tetrahydroxyxanthone 8-prenyltransferase*, were consistently upregulated after both treatments. In contrast, genes central to flavonoid biosynthesis, such as *chalcone synthase* (*CHS*) and *flavonol synthase* (*FLS*), were downregulated (Fig. 6).

## Discussion

### Agrobacterium infection activates pattern recognition receptors and signal transduction cascades

The plant immune system depends entirely on pattern recognition receptors (PRRs), which recognize both foreign and plant-derived molecular patterns to signal potential health threats [30]. Upon treatment with both *Agrobacterium* species, plant-derived receptors such as *leucine-rich repeat (LRR) receptor-like serine/threonine kinase* (*At1g74360*), *LysM domain receptor-like kinase 4 (LYK4)* and *G-type lectin S-receptor-like serine/threonine-protein kinase (CES101)* were activated, serving as early sensors for pathogen-associated molecular patterns (PAMPs) (Fig. 4A). In maize, the *fungal-induced receptor-like leucine-rich repeat protein kinase (FI-RLPK)* functions as a pattern recognition receptor that mediates jasmonic acid-dependent production of antimicrobial phytoalexins in response to fungal pathogens and contributes to disease resistance [31]. In another study, overexpression of *LYK4* increased disease resistance not only in leaves but also in fruits, suggesting that increased expression of this LysM receptor-like kinase contributes to chitin-induced immunity and plays an important role in protecting plants against fungal pathogens such as *Botrytis cinerea* [32]. Interestingly, *CES101* has also been reported as an early and strongly induced gene in anthracnose-resistant walnut (*Juglans regia*), where its expression induced immediately after *Colletotrichum gloeosporioides* infection, while susceptible genotypes showed delayed induction [33]. The upregulation of *salicylic acid-binding protein 2 (SABP2)* observed in our study is consistent with previous findings that overexpression of *SABP2* in citrus enhances systemic acquired resistance and effectively suppresses the proliferation of the Gram-negative bacterium *Candidatus* Liberibacter asiaticus [34], suggesting that it plays a potential role in enhancing defense signals following *Agrobacterium* treatment.

### Oxidative stress and ros-scavenging pathways were activated following Agrobacterium treatment

Genes such as the respiratory burst oxidase homologue (RBOH) were induced after treatment with both *Agrobacterium* species, facilitating ROS burst, a common feature of innate immunity in plants. ROS play a dual role by directly attacking pathogens and serving as signaling molecules to enhance downstream defenses [35]. Antioxidants play a critical role in mitigating oxidative damage caused by ROS bursts during pathogen attack [36]. Several genes implicated in ROS scavenging such as *glutathione S-transferase U7 (GSTU7)*,* peroxidase N (PER N)*,* peroxidase N1 (PER N1)* and *peroxidase 21 (PER 21)*, were strongly upregulated after *Agrobacterium* treatment (Fig. 4A). The strong upregulation of *GSTU7* in *H. perforatum* following *Agrobacterium* infection suggests an important role in attenuating oxidative stress and is consistent with findings from *Arabidopsis thaliana* where, *GSTU7* regulated by class II TGA transcription factors, acts as a glutathione peroxidase that limits ROS accumulation, preserves glutathione redox homeostasis, and enhances stress resilience under oxidative conditions [37]. Studies show that members of the class III peroxidase family contribute substantially to oxidative stress responses by maintaining ROS homeostasis through localized scavenging activity, especially under biotic and abiotic stress conditions [38] further supporting the overexpression of peroxidases in our study.

### Stress responsive transcription factors were induced during immune activation in H. perforatum

Transcription factors (TFs) act as molecular switches that control gene expression in response to pathogen attack. In this study, transcriptomic data revealed the activation of numerous TF families upon treatment with *A. tumefaciens* as well as *A. rhizogenes*. Several WRKY members (e.g. *WRKY24*, *WRKY33*,* WRKY72*) were significantly induced (Fig. 4B). These are known regulators of stress responses, particularly in the modulation of defense mechanisms such as salicylic acid (SA). *WRKY33*, for example, is associated with resistance to necrotrophic pathogens [39] and may play a role in the antimicrobial responses of *H. perforatum*. TFs of the MYB family, including *MYB4* and *MYB15*-like, have been activated. *MYB4* is often associated with the suppression of flavonoid biosynthesis, which is consistent with the observed downregulation of *CHS* in favor of xanthone production [40]. *MYB15*-like factors are also known to mediate responses to environmental stress, including pathogen attack [41]. Ethylene-responsive transcription factors (ERF) such as *ERF113* have been induced, suggesting that they play a role in the activation of genes associated with ethylene-mediated defense responses, such as proteins related to pathogenesis. ERFs often function in tandem with other hormonal pathways to fine-tune the immune response [42]. Zinc finger proteins such as *ZAT10* and basic helix-loop-helix TFs (e.g. *bHLH92*) have also been shown to be upregulated and contribute to the regulation of oxidative stress responses and defense-related biosynthesis of secondary metabolites [43].

### Defense effectors were strongly activated following Agrobacterium infection

The plant’s defense mechanism was clearly activated after exposure to both *Agrobacterium* species. Members of the PR10 family were strongly upregulated. These proteins are multifunctional and contribute to antimicrobial activity, RNA cleavage and cell wall remodelling. For example, PR-10 proteins are known to interact with fungal and bacterial pathogens by degrading their RNA or inhibiting their growth [44]. Consistent with our observation that *PR4* is induced in *H. perforatum* cells following *Agrobacterium* treatment, recent studies have shown that transient overexpression of *PR4A* from citrus significantly increases resistance to *Xanthomonas citri* in sweet orange. Moreover, transgenic *Arabidopsis* lines overexpressing *PR4A* exhibit improved resistance to *Pseudomonas syringae* pv. tomato DC3000, emphasizing the conserved defensive role of PR4 proteins [45]. LOXs, such as *linoleate 9S-lipoxygenase 5*, were activated (Fig. 4B) and further contributed to the synthesis of oxylipins such as jasmonic acid, which are crucial for plant defense signaling and antimicrobial activities [46].

### Upregulation of xanthone biosynthetic genes accompanied downregulation of flavonoid pathway

Secondary metabolites play a central role in plant defense by acting as antimicrobial agents and antioxidants [47]. Key genes of xanthone pathway, such as *BPS* and *1*,*3*,*7-trihydroxyxanthone synthase* (*CYP81AA*), were significantly upregulated (Fig. 4C). The characterization of BPS from *Garcinia mangostana* underlines the substrate flexibility of the enzyme and its central role in the initiation of xanthone biosynthesis [48]. Functional elucidation of *benzoyl coenzyme A ligase* (*BZL*), *BPS* and *benzophenone 3′-hydroxylase* (*B3*′H) from *Cudrania tricuspidata* reveals a stepwise xanthone biosynthetic pathway in which *BZL* converts benzoate to benzoyl-CoA, *BPS* catalyzes its condensation with malonyl-CoA to generate 2,4,6-trihydroxybenzophenone, and *B3′H* hydroxylates this intermediate to form 2,3′,4,6-tetrahydroxybenzophenone, an important precursor for xanthone biosynthesis [49]. Up-regulation of *CYP81AA* catalyzes the oxidative cyclisation of tetrahydroxybenzophenone to 1,3,7-trihydroxyxanthone, a central scaffold for several xanthone derivatives such as gentisin, hyperixanthone A, α-mangostin and mangiferin, as has been reported in several plant families including the Hypericaceae [50]. The overexpression of *PAL* in our study highlights its central role as a gateway enzyme of the phenylpropanoid pathway, similar to the observations in *Peucedanum praeruptorum*, where functionally distinct *PAL* isoforms catalyze the conversion of L-phenylalanine to trans-cinnamic acid, a crucial step that modulates the downstream biosynthesis of phenolics and defense-related secondary metabolites [51].

Interestingly, several central genes of flavonoid biosynthesis, including 4*-coumarate-CoA ligase1* (*4CL1*), *CHS* and *FLS*, were downregulated in our study. *4CL1* catalyzes the conversion of 4-coumaric acid to 4-coumaroyl-CoA, an important intermediate in the phenylpropanoid pathway [52], and its reduced expression likely limits flux into downstream flavonoid branches. *CHS*, a type III polyketide synthase, condenses 4-coumaroyl-CoA with malonyl-CoA to generate naringenin chalcone, the first crucial step in flavonoid biosynthesis that serves as a critical checkpoint for flux in the flavonoid pathway [53, 54]. Thus, downregulation of *CHS* indicates a redirection of metabolic resources away from flavonoid production and possibly toward increased xanthone biosynthesis to enhance antimicrobial defense. Similarly, *FLS*, which catalyzes the conversion of dihydroflavonols to flavonols, regulates plant growth, development, and response to environmental stress [55]; its repression implies a strategic attenuation of flavonol-mediated stress adaptation in favor of xanthone-driven prioritization of defense under *Agrobacterium*-induced stress conditions.

### Downregulation of flavonoid genes coincided with targeted accumulation of antimicrobial metabolites

Although transcriptomic analysis revealed downregulation of *CHS* and *4CL*, key enzymes of the flavonoid biosynthetic pathway, the significant accumulation of procyanidin B II, a flavonoid, suggests that flavonoid production is not completely repressed but may be regulated post-transcriptionally or via alternative branches of the phenylpropanoid pathway. B-type procyanidins, including procyanidin B II, are known for their broad-spectrum antibacterial activity. This has been demonstrated in extracts of lotus seedpod, which showed strong inhibition of *Escherichia coli* with minimal inhibitory concentrations as low as 1.25 mg/mL [56]. Remarkably, among the major constituents, procyanidin dimers such as B1 and B2 showed measurable antibacterial effects, supporting their potential role in defense mechanisms against bacterial pathogens. Procyanidins from larch bark (LBPCs) exert potent antibacterial effects by interfering with the integrity of bacterial cell wall and membrane, disrupting protein synthesis, impairing energy metabolism, and binding to DNA, thereby effectively inhibiting the growth of *Staphylococcus aureus* [57]. The antimicrobial activity of procyanidins from açaí seeds, particularly against *S. aureus* and *Candida albicans*, is attributed to combined mechanisms, such as disruption of bacterial membranes by high-degree polymers and inhibition of enzymes or chelation of metal ion by oligomers [58].

The upregulation of *PAL* suggests an increased flux into the general phenylpropanoid pathway, which may serve as a precursor for xanthone biosynthesis and selected flavonoid subclasses. The concomitant upregulation of *BPS* suggests that metabolic intermediates are redirected to the production of xanthones, a class of antimicrobial and antioxidant phenolics, rather than conventional flavonoids. This shift probably reflects a strategic metabolic redistribution that favors the accumulation of xanthones and specific flavonoids (e.g., procyanidin B II) that are more effective against pathogenic oxidative and microbial stress.

### Agrobacterium exposure induced the production of antimicrobial xanthones

Metabolomics results emphasize the role of phenolic compounds in combating oxidative stress induced by pathogen attack [59]. The accumulation of xanthones in the present study supports the view of their function in antimicrobial defense and provides insights into the biochemical strategies employed by *H. perforatum* against *Agrobacterium* species. Comparable responses have been reported in cell suspensions of *H. perforatum* elicited with *A. tumefaciens* and *A. rhizogenes*, which showed markedly increased levels of phenolics and xanthones [19]. These findings are consistent with our earlier findings, where *H. perforatum* cultures co-cultivated with *A. tumefaciens* exhibited a 12-fold increase in total xanthone content and the de novo synthesis of xanthone derivatives with potent antioxidant and antimicrobial properties—underscoring the dual role of xanthones as ROS-scavenging antioxidants and phytoalexins in biotic stress [60]. Recent studies have highlighted xanthone derivatives, such as compound XT17, as potent broad-spectrum antibacterial agents that exert multifaceted mechanisms of action, including disruption of bacterial cell walls and inhibition of DNA synthesis [61]. Xanthones isolated from Calophyllum species have shown potent antibacterial activity, primarily attributed to their ability to inhibit bacterial DNA gyrase and topoisomerase IV, key enzymes of nucleic acid synthesis, as demonstrated through molecular docking studies [62]. Isojacareubin, a xanthone isolated from *Hypericum japonicum*, has demonstrated potential activity against methicillin-resistant *Staphylococcus aureus* and synergistic effects with conventional antibiotics, underscoring its potential as a novel antimicrobial agent [63]. Interestingly, in our study, isojacareubin was found to be upregulated in *H. perforatum* following *Agrobacterium* treatment, suggesting a possible role in defense against bacterial colonization, which is consistent with its reported antibacterial mechanism.

### Agrobacterium species triggered similar defense pathways in H. perforatum

We used two different species of *Agrobacterium*, *A. tumefaciens* and *A. rhizogenes*, to investigate their transcriptomic and metabolomic effects on *H. perforatum*. Several strains of *A. tumefaciens* and *A. rhizogenes* had been shown to be quite ineffective in transforming *H. perforatum* in our previous studies, suggesting that the species as a whole is resistant to transformation by these bacteria [1]. Nevertheless, some studies have indicated successful development of hairy roots by *A. rhizogenes* in Hypericum species, although the efficiency of transformation varies depending on the strain [64–66].

Differences in the susceptibility of different Hypericum species and explant types to different *A. rhizogenes* strains have also been observed. For instance, a previous report showed a greater frequency of transformation of *H. tetrapterum* explants with strain 15834 (73%) compared to strain A4 (13%). The same publication also reported that roots formed hairy root cultures successfully, while leaf explants remained resistant to *A. rhizogenes* [64].

In the present study, treatments with both *Agrobacterium* species resulted in substantial transcriptomic and metabolomic regulation. Treatment with *A. rhizogenes* induced a greater number of differentially expressed genes and more accumulated metabolites than treatment with *A. tumefaciens*. In contrast to these differences, the overall pattern of change in gene expression was similar between the two treatments (Table S2), suggesting that both species induce plant defense mechanisms by similar mechanisms.

Taken together, these results support the hypothesis that antimicrobial secondary metabolites, such as xanthones and specific flavonoids, including procyanidin B II, contribute substantially to the innate immune response of *H. perforatum* by impairing the viability of Agrobacterium and limiting its ability to colonize successfully. Our results provide comparative data on the host response to *Agrobacterium* treatment of *H. perforatum*.

## Conclusion

This is the first study to provide a combined transcriptomic and metabolomic insight into the molecular basis of transformation resistance in *H. perforatum*. It provides compelling molecular evidence for the recalcitrance of *H. perforatum* to *Agrobacterium*-mediated transformation by revealing an early, multi-step defense response. Using an integrated transcriptomic and metabolomic approach, we demonstrated that *H. perforatum* recognizes *Agrobacterium* through an early induction of receptor-like kinases and defense-related transcription factors. One of the most important effects is the targeted reprogramming of phenylpropanoid metabolism, which suppresses flavonoid biosynthesis and strongly promotes the xanthone branch. This is supported by the concerted upregulation of *BPS* and *CYP81AA* and the accumulation of bioactive xanthones such as 6-deoxyisojacareubin and hyperxanthone E with antimicrobial activity. These metabolites, in combination with transcriptional reprogramming, create a hostile cellular environment that should limit colonization and transformation by *Agrobacterium*. By identifying key genes and metabolic shifts responsible for early pathogen recognition and defense activation, particularly the strong metabolic redirection towards antimicrobial xanthones, this work provides important targets for future manipulations. This dataset lays a solid foundation for the development of novel strategies for *Agrobacterium*-mediated transformation and recombinant gene editing in *H. perforatum*, which could greatly expand its utility for metabolic engineering and functional genomics.

## Supplementary Information

Supplementary Material 1.

Supplementary Material 2.

Supplementary Material 3.

## Data Availability

The transcriptomic data generated in this study are publicly available at ArrayExpress under the accession number E-MTAB-15161 (https://www.ebi.ac.uk/biostudies/arrayexpress/studies/E-MTAB-15161).
