# Supplementary material for: Dual omics comparison: how Agrobacterium tumefaciens and Agrobacterium rhizogenes modulate gene expression and metabolism in Hypericum perforatum L
Source: BMC Genomics. 2025 Oct 24;26:958. doi: 10.1186/s12864-025-12086-8 (PMC12553202; doi:10.1186/s12864-025-12086-8)
Supplement: Supplementary file 2 — Supplementary Material 2. [file 12864_2025_12086_MOESM2_ESM.docx]

Supplementary information

**Dual Omics Comparison: How *Agrobacterium tumefaciens* and *Agrobacterium* *rhizogenes* Modulate Gene Expression and Metabolism in *Hypericum perforatum***

Rajendran K. Selvakesavan, Maria Nuc, Matam Pradeep, Paweł Krajewski*, Gregory Franklin*

Institute of Plant Genetics of the Polish Academy of Sciences, Strzeszyńska 34, 60-479

Poznań, Poland

| 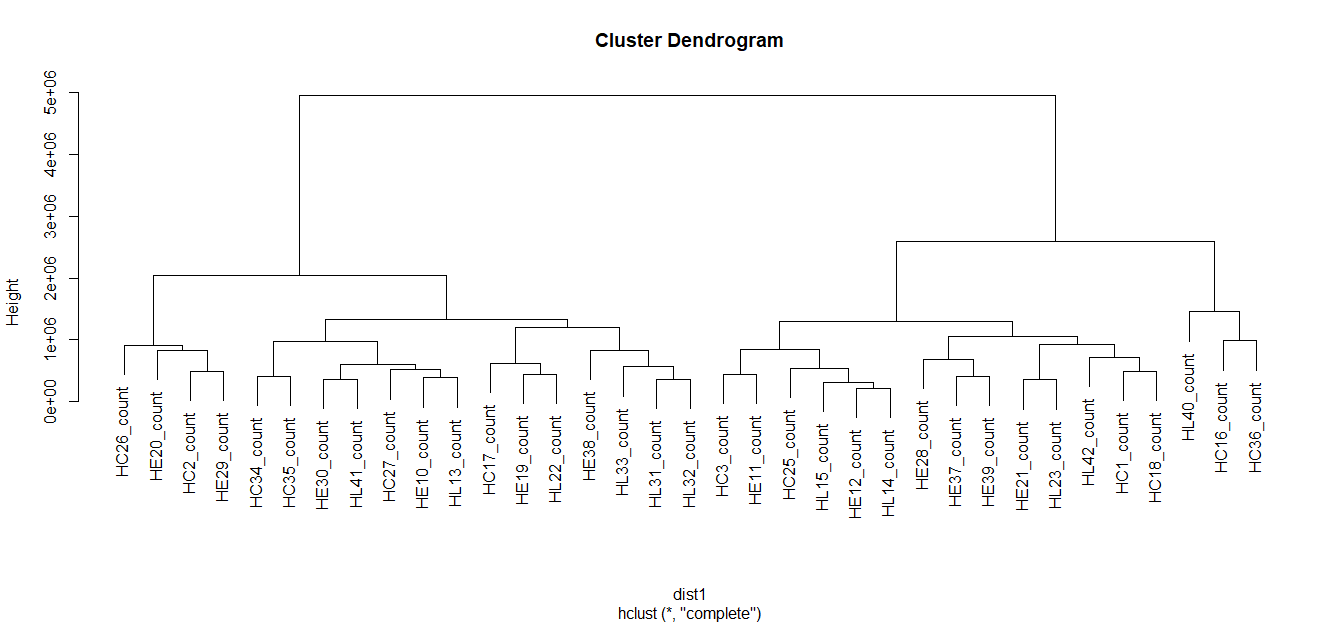 |
| --- |
| Fig. S1. Hierarchical clustering of the RNA-seq samples based on the number of reads mapped to 88 616 reference transcripts of *H. perforatum* (Euclidean distance matrix, complete link clustering method); see Supplementary Table 1 for the description of the IDs of the samples. |

A

|  | Sample IDs |
| --- | --- |
| Sample number | 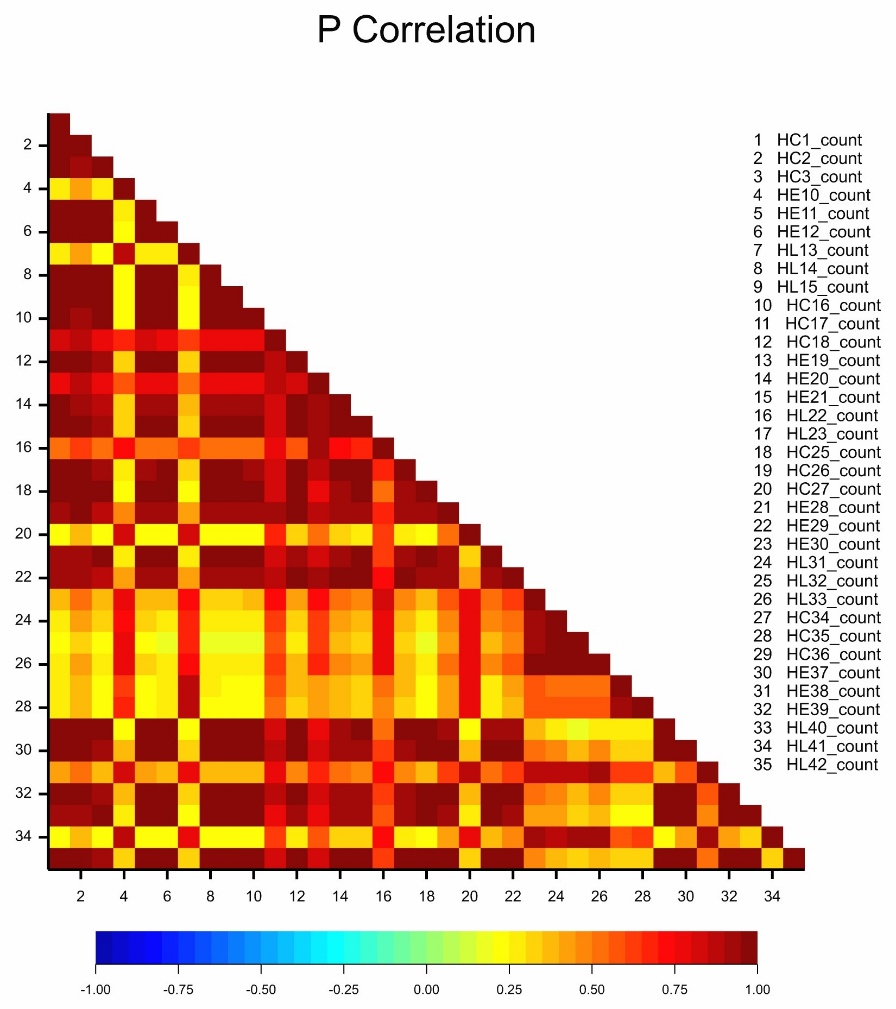 |
|  | Pearson correlation coefficient |

B

| Variant number | Treatment | Time (hours) | Samples | Correlation between replications | |
| --- | --- | --- | --- | --- | --- |
|  |  |  |  | Minimum | Maximum |
| 1 | Control | 0.5 | 1, 2, 3 | 0.9306 | 0.9810 |
| 2 | *A. tumefaciens* | 0.5 | 4, 5, 6 | **0.2412** | 0.9894 |
| 3 | *A. rhizogenes* | 0.5 | 7, 8, 9 | **0.2304** | 0.9964 |
| 4 | Control | 4 | 10, 11, 12 | 0.8000 | 0.9695 |
| 5 | *A. tumefaciens* | 4 | 13, 14, 15 | 0.9035 | 0.9953 |
| 6 | *A. rhizogenes* | 4 | 16, 17 | 0.6876 | 0.6876 |
| 7 | Control | 12 | 18, 19, 20 | **0.2374** | 0.9349 |
| 8 | *A. tumefaciens* | 12 | 21, 22, 23 | 0.5125 | 0.9477 |
| 9 | *A. rhizogenes* | 12 | 24, 25, 26 | 0.9693 | 0.9899 |
| 10 | Control | 24 | 27, 28, 29 | **0.2680** | 0.9388 |
| 11 | *A. tumefaciens* | 24 | 30, 31, 32 | 0.5842 | 0.9964 |
| 12 | *A. rhizogenes* | 24 | 33, 34, 35 | **0.3132** | 0.9567 |

Fig. S2. A. Heatmap visualization of Pearson correlation coefficients between read counts in all samples. B. Minimum and maximum correlation coefficients between read counts in samples representing experimental variants (treatment × time combinations). See Supplementary Table 1 for explanation of sample IDs.

| A | B |
| --- | --- |
| 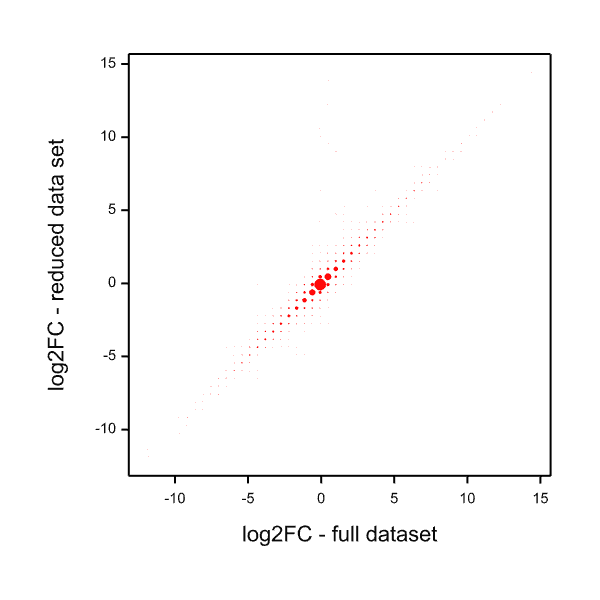 | 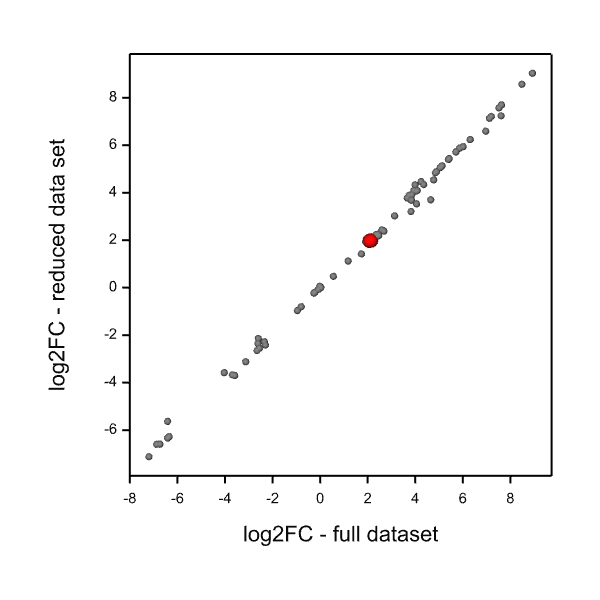 |
|  |  |

Fig. S3. Results of sensitivity analysis for RNA-seq data – comparison of differential gene expression analysis for full dataset (35 replicates) and a reduced dataset obtained by excluding 5 replicates with low correlations with other replications within experimental variant. A. The density plot of log2FC values for all 8 tested contrasts between treated and control samples, for all 88616 transcripts, obtained using full dataset (X axis) v. values obtained using the reduced dataset (Y axis); computed Pearson correlation coeffcient equal to 0.984. B. Plot as in A but only for log2FC values obtained for 9 transcripts considered as mostly important for drawing conclusions about xanthone and flavonoid biosynthesis pathways (transcript_c_6220, transcript_c_21290, transcript_c_15382, transcript_c_11985, transcript_c_25557, transcript_s_27615, transcript_c_27516, transcript_c_20109, transcript_c_25667). Red dot indicates two (very close) points corresponding to situations, in which contrasts were found significant in the analysis of full dataset but not significant in the analysis done for reduced dataset because of the log2FC values in the latter analysis were slightly lower than 2 (1.99 and 1.97).


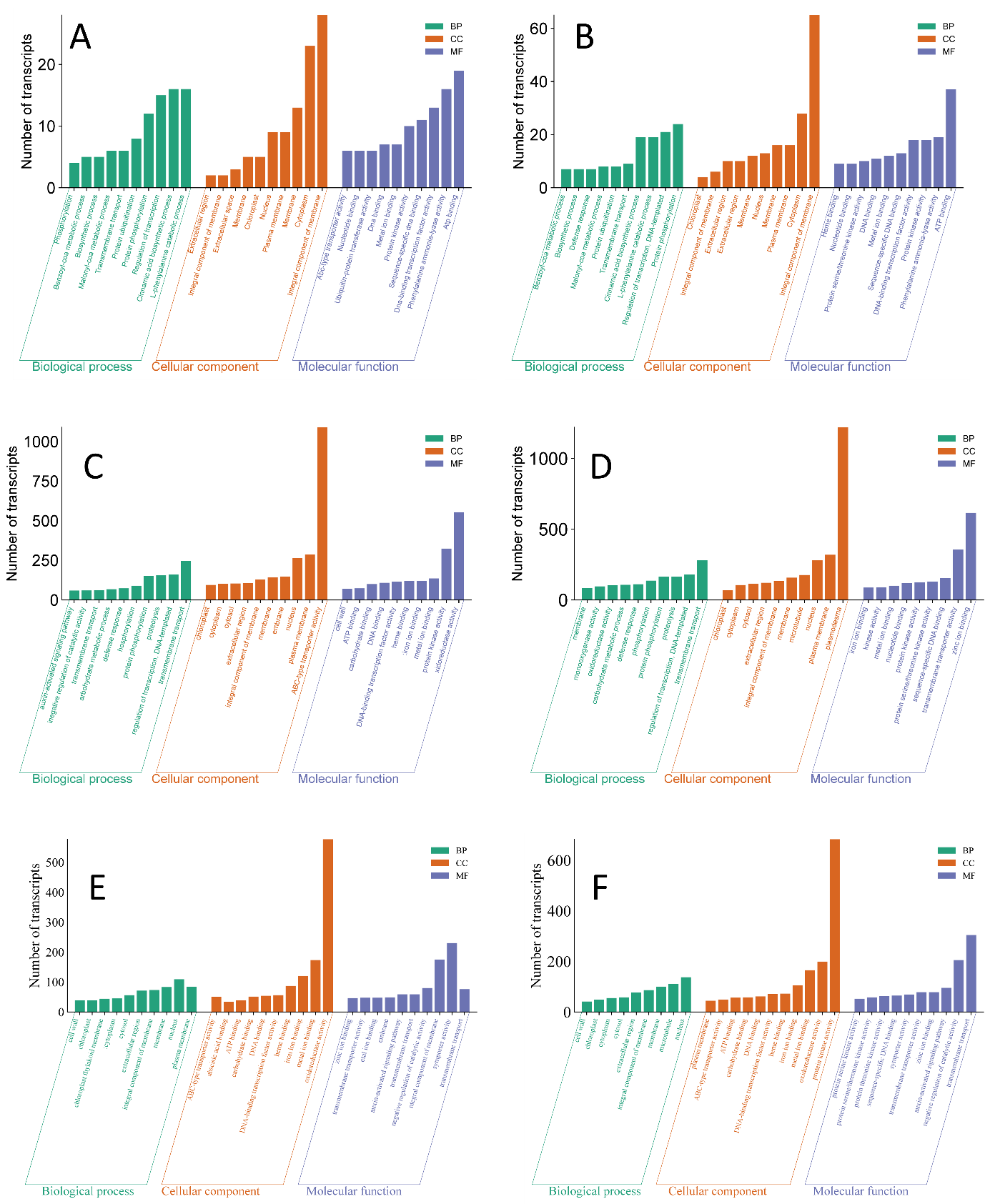


Fig. S4. Histogram of GO function analysis for differentially expressed genes after Agrobacterium treatment (A. A. tumefaciens (0.5 h); B. *A. rhizogenes* (0.5 h); C. *A. tumefaciens* (12 h); D. *A. rhizogenes* (12 h)); E. *A. tumefaciens* (24 h); F. *A. rhizogenes* (24 h));The top ten GO terms with the maximum number of transcripts are displayed for each classification.

Table S3. Gene symbols and corresponding full gene names of transcripts used for heat map analysis in Fig. 4.

| **Gene symbol** | **Gene Name** | **Transcript Number** |
| --- | --- | --- |
| *At1g74360* | *Probable LRR receptor-like serine/threonine-protein kinase At1g74360* | transcript_c_4504 |
| *At1g53430* | *probable LRR receptor-like serine/threonine-protein kinase At1g53430* | transcript_c_5062 |
| *LYK4* | *LysM domain receptor-like kinase 4* | transcript_c_15569 |
| *LRK10L-2.1* | *Leaf Rust 10 Disease-Resistance Locus Receptor-Like Protein Kinase-like 2.1* | transcript_c_20257 |
| *LRK10* | *Rust resistance kinase Lr10-like* | transcript_c_12090 |
| *CES101* | *G-type lectin S-receptor-like serine/threonine-protein kinase CES101 isoform X3* | transcript_c_9795 |
| *RIPK* | *Serine/threonine-protein kinase RIPK-like* | transcript_c_23884 |
| *CRLK1* | *Calcium/calmodulin-regulated receptor-like kinase 1* | transcript_s_24426 |
| *SABP2* | *Salicylic acid-binding protein 2* | transcript_c_29267 |
| *CRK2* | *Cysteine-rich receptor-like protein kinase 2* | transcript_c_12420 |
| *RPK2* | *Receptor like protein kinase S.2* | transcript_s_10428 |
| *GLR2.1* | *glutamate receptor 2.1* | transcript_s_2061 |
| *GSO1* | *LRR receptor-like serine/threonine-protein kinase GSO1* | transcript_s_5512 |
| *WAKL10* | *Wall-associated receptor kinase-like 10* | transcript_s_15135 |
| *CRCK2* | *Calmodulin-binding receptor-like cytoplasmic kinase 2* | transcript_s_34616 |
| *AT4G39830/AO* | *L-ascorbate oxidase homolog* | transcript_s_22682 |
| *GSTU7* | *Glutathione S-transferase U7* | transcript_c_20540 |
| *AIFM2* | *ferroptosis suppressor protein 1* |  |
| *PER N* | *Peroxidase N* | transcript_c_18245 |
| *PER21* | *Peroxidase 21-like* | transcript_s_45551 |
| *PERN1* | *Peroxidase N1* | transcript_c_26990 |
| *ERF113* | *Ethylene-responsive transcription factor ERF113* | transcript_c_28495 |
| *ZAT10* | *Zinc finger protein ZAT10* | transcript_c_28494 |
| *SCL14* | *Scarecrow-like protein 14* | transcript_c_9921 |
| *WRKY72* | *Probable WRKY transcription factor 72* | transcript_s_30374 |
| *WRKY33* | *Probable WRKY transcription factor 33* | transcript_c_20293 |
| *WRKY24* | *WRKY transcription factor WRKY24-like* | transcript_c_14911 |
| *bHLH92* | *Transcription factor bhlh92* | transcript_c_27870 |
| *MYB78* | *Transcription factor MYB78-like* | transcript_c_26022 |
| *MYB15* | *Transcription factor MYB15-like* | transcript_c_27820 |
| *MYB4* | *Transcription factor MYB4-like* | transcript_c_29821 |
| *ABR1* | *Ethylene-responsive transcription factor ABR1-like* | transcript_c_24377 |
| *PR-4* | *Pathogenesis-related protein PR-4* | transcript_c_31934 |
| *PR-10* | *PR-10 protein* | transcript_c_30538 |
| *PPO* | *Polyphenol oxidase, chloroplastic-like* | transcript_s_20681 |
| *LOX1.5* | *Probable linoleate 9S-lipoxygenase 5* | transcript_s_6572 |
| *ABCG* | *ABC transporter G* | transcript_c_1719 |
| *RBOHD* | *Respiratory burst oxidase homolog protein D* | transcript_c_16323 |
| *GSTL3* | *Glutathione S-transferase L3-like isoform X1* | transcript_c_9712 |
| *GIP2* | *Probable aspartic proteinase GIP2* | transcript_s_47445 |
| *PDR1* | *Pleiotropic drug resistance protein 1* | transcript_c_4212 |
| *PTpat* | *8-prenyl-1,3,6,7-tetrahydroxyxanthone 8-prenyltransferase* | transcript_c_6220 |
| *UGT73C3* | *UDP-glycosyltransferase 73C3* | transcript_c_21611 |
| *CYP81AA* | *1,3,7-trihydroxyxanthone synthase* | transcript_c_21290 |
| *FLS* | *Flavonol synthase* | transcript_c_25667 |
| *COMT* | *Caffeic acid 3-O-methyltransferase-like* | transcript_c_25666 |
| *MO2* | *Monooxygenase 2* | transcript_c_5147 |
| *PAL* | *Phenylalanine ammonia-lyase* | transcript_c_11985 |
| *CHS* | *Chalcone synthase* | transcript_c_27516 |
| *BPS* | *Benzophenone synthase* | transcript_c_25557 |
| *UF3GT* | *UDP-glycose flavonoid 3-O-glycosyltransferase* | transcript_s_41310 |
| *At4g20840* | *Berberine bridge enzyme-like 21* | transcript_c_11971 |
| *CYP71A9* | *Cytochrome P450 71A9* | transcript_c_11746 |
| *ROMT* | *Trans-resveratrol di-O-methyltransferase-like* | transcript_c_22689 |
| *SAM5* | *S-adenosylmethionine synthase 5* | transcript_s_13855 |
| *CNL* | *Trans-cinnamate:coa ligase, peroxisomal-like* | transcript_s_27615 |
| *BAS* | *Beta-amyrin synthase* | transcript_c_8174 |
| *LOC_Os07g09190* | *Probable 1-deoxy-D-xylulose-5-phosphate synthase 2, chloroplastic* | transcript_c_12007 |
| *ISPH* | *4-hydroxy-3-methylbut-2-enyl diphosphate reductase, chloroplastic* | transcript_c_17292 |
| *LOC107925105* | *Casbene synthase, chloroplastic-like* | transcript_c_19780 |
| *4CLL1* | *4-coumarate--CoA ligase 1-like* | transcript_c_20109 |

* Transcript Number corresponding to Table S2

Table S4. Comprehensive list of differentially accumulated secondary metabolites detected in *H. perforatum* cells in response to *A. tumefaciens* and *A. rhizogenes* treatment.

| S.No. | Compound name | Control  (mg/g DW) | | *A. tumefaciens*  (mg/g DW) | | *A. rhizogenes*  (mg/g DW) | |
| --- | --- | --- | --- | --- | --- | --- | --- |
|  |  | 30 min | 4 h | 30 min | 4 h | 30 min | 4 h |
| 01 | Hydroxybenzoic acid hexoside I | 0.16±0.01 | 0.16±0.01 | 0.13±0.01 | 0.15±0.01 | 0.15±0.01 | 0.15±0.01 |
| 02 | Dihydroxybenzoic acid hexoside | 0.24±0.01 | 0.17±0.001 | 0.2±0.01 | 0.16±0.001 | 0.23±0.01 | 0.17±0.01 |
| 03 | Hydroxybenzoic acid hexoside II | 0.51±0.02 | 0.75±0.1 | 0.4±0.02 | 0.48±0.01 | 0.49±0.01 | 0.43±0.02 |
| 04 | Hypericophenonoside I | 0 | 0 | 0 | 0 | 0 | 0 |
| 05 | Isotachioside | 0.06±0.003 | 0.05±0.001 | 0.05±0.01 | 0.04±0.01 | 0.04±0.02 | 0.05±0.001 |
| 06 | Caffeic acid hexoside | 0.36±0.02 | 0.34±0.02 | 0.27±0.02 | 0 | 0.31±0.01 | 0 |
| 07 | Coumaroylquinic acid | 0.29±0.01 | 0.27±0.01 | 0.23±0.001 | 0 | 0.25±0.01 | 0 |
| 08 | Quercetin dihexoside | 0.28±0.02 | 0.25±0.01 | 0.22±0.02 | 0.23±0.01 | 0.25±0.01 | 0.23±0.01 |
| 09 | (Epi)catechin | 1.13±0.06 | 1.16±0.04 | 0.93±0.07 | 0.99±0.04 | 1.12±0.04 | 1.06±0.05 |
| 10 | Maclurin | 0.09±0.01 | 0.03±0.03 | 0.05±0.02 | 0.09±0.001 | 0.01±0.001 | 0.01±0.001 |
| 11 | Trihydroxybenzophenone O/C-hexoside | 0.22±0.01 | 0.21±0.01 | 0.17±0.01 | 0.19±0.01 | 0.19±0.001 | 0.18±0.01 |
| 12 | Procyanidin C | 0.18±0.01 | 0.19±0.01 | 0.16±0.02 | 0.19±0.001 | 0.17±0.001 | 0.17±0.01 |
| 13 | Mangiferin | 0.13±0.01 | 0.13±0.001 | 0.11±0.001 | 0.12±0.01 | 0.11±0.001 | 0.2±0.04 |
| 14 | Kaempferol hexoside | 1.36±0.05 | 1.52±0.08 | 1.25±0.05 | 1.42±0.09 | 1.3±0.04 | 1.26±0.1 |
| 15 | Procyanidin B II | 0 | 0 | 0 | 0 | 0 | 0 |
| 16 | Quercetin hexoside | 1.83±0.07 | 1.92±0.06 | 1.53±0.08 | 1.77±0.12 | 1.74±0.08 | 1.77±0.07 |
| 17 | Quercetin acetylhexoside | 0.95±0.03 | 0.92±0.01 | 0.78±0.07 | 0.9±0.06 | 0.91±0.04 | 0.9±0.04 |
| 18 | TetraOH,MeOxanthone hexoside | 0 | 0 | 0 | 0 | 0 | 0 |
| 19 | Neolancerin | 0.06±0.002 | 0.08±0.03 | 0.07±0.01 | 0.06±0.001 | 0.06±0.001 | 0.06±0.001 |
| 20 | DiHO,diMeOxanthone 1 | 0.17±0.005 | 0.16±0.01 | 0.14±0.01 | 0.16±0.01 | 0.16±0.01 | 0.15±0.01 |
| 21 | TtriHO,MeOxanthone malonylhexoside | 0.15±0.003 | 0.13±0.01 | 0.12±0.01 | 0.13±0.01 | 0.13±0.001 | 0.13±0.001 |
| 22 | DiOH,diMeOxanthone hexoside | 0.24±0.005 | 0.21±0.01 | 0.19±0.02 | 0.23±0.01 | 0.21±0.01 | 0.24±0.02 |
| 23 | Tetra-hydroxy-xanthone | 0 | 0 | 0 | 0 | 0 | 0 |
| 24 | TetraHO,MeOxanthone | 0.06±0.001 | 0.04±0.001 | 0.04±0.001 | 0.05±0.001 | 0.05±0.001 | 0.04±0.001 |
| 25 | DiMeO,Hoxanthone | 0.12±0.01 | 0.04±0.02 | 0.1±0.03 | 0.08±0.01 | 0.09±0.01 | 0.06±0.01 |
| 26 | Bisxanthone | 0 | 0 | 0 | 0 | 0 | 0 |
| 27 | Hyperxanthone B | 0 | 0 | 0 | 0.05±0.01 | 0 | 0.04±0.01 |
| 28 | TriHO,diMeOxanthone | 0.03±0.01 | 0.01±0.001 | 0.02±0.001 | 0.01±0.001 | 0.02±0.001 | 0.01±0.001 |
| 29 | TriHO,MeOxanthone 1 | 0.06±0.004 | 0.03±0.01 | 0.04±0.01 | 0.06±0.001 | 0.05±0.001 | 0.04±0.001 |
| 30 | Hyperxanthone A | 0.09±0.005 | 0.04±0.001 | 0.07±0.01 | 0.05±0.001 | 0.08±0.001 | 0.04±0.001 |
| 31 | Cadensin G | 0.19±0.006 | 0.16±0.001 | 0.17±0.01 | 0.17±0.001 | 0.18±0.001 | 0.16±0.001 |
| 32 | DiHO,diMeOxanthone 3 | 0.04±0.007 | 0.01±0.001 | 0.02±0.001 | 0 | 0.03±0.001 | 0 |
| 33 | Hyperxanthone C | 0 | 0 | 0 | 0 | 0 | 0 |
| 34 | DiHO,diMeOxanthone 4 | 0 | 0 | 0 | 0 | 0 | 0 |
| 35 | Gemixanthone A | 0 | 0 | 0 | 0 | 0 | 0 |
| 36 | Toxyloxanthone B | 0 | 0 | 0 | 0 | 0 | 0 |
| 37 | Hyperxanthone E | 0 | 0 | 0 | 0.02±0.001 | 0 | 0.02±0.01 |
| 38 | Gancaonin O | 0 | 0 | 0 | 0 | 0 | 0 |
| 39 | 2-deprenyl-7-HO-rheediaxanthone | 0.02±0.001 | 0.03±0.01 | 0.03±0.001 | 0.05±0.01 | 0.03±0.01 | 0.04±0.001 |
| 40 | Hyperxanthone D | 0.01±0.001 | 0 | 0.01±0.001 | 0.01±0.001 | 0.01±0.001 | 0.01±0.001 |
| 41 | Paxanthone | 0.05±0.004 | 0.05±0.001 | 0.04±0.001 | 0.04±0.001 | 0.05±0.001 | 0.04±0.001 |
| 42 | TriHO,MeOprenylxanthone 4 | 0.2±0.005 | 0.2±0.001 | 0.19±0.001 | 0.2±0.001 | 0.19±0.001 | 0.19±0.001 |
| 43 | 6-deoxyisojacareubin | 0 | 0 | 0 | 0 | 0 | 0 |
| 44 | DiHO,diMeOprenylxanthone | 0 | 0 | 0 | 0 | 0 | 0 |
| 45 | Isojacareubin | 0.01±0.001 | 0.01±0.001 | 0.01±0.001 | 0.01±0.001 | 0.01±0.001 | 0.01±0.001 |
| 46 | Gamma-Mangostin | 0.01±0.001 | 0.01±0.001 | 0.01±0.001 | 0.02±0.001 | 0.01±0.001 | 0.01±0.001 |
| 47 | Garcinone B | 0.01±0.004 | 0.02±0.01 | 0.01±0.001 | 0.01±0.001 | 0.01±0.001 | 0 |
| 48 | Roeperanone | 0 | 0 | 0 | 0 | 0 | 0 |

| S.No. | Compound name | Control  (mg/g DW) | | *A. tumefaciens*  (mg/g DW) | | *A. rhizogenes*  (mg/g DW) | |
| --- | --- | --- | --- | --- | --- | --- | --- |
|  |  | 12 h | 24 h | 12 h | 24 h | 12 h | 24 h |
| 01 | Hydroxybenzoic acid hexoside I | 0.17±0.001 | 0.18±0.01 | 0.19±0.04 | 0.25±0.01 | 0.23±0.01 | 0.29±0.01 |
| 02 | Dihydroxybenzoic acid hexoside | 0.19±0.001 | 0.23±0.01 | 0.16±0.03 | 0.29±0.05 | 0.2±0.01 | 0.4±0.01 |
| 03 | Hydroxybenzoic acid hexoside II | 0.66±0.01 | 0.61±0.03 | 0.37±0.08 | 0.09±0.03 | 0.45±0.01 | 0 |
| 04 | Hypericophenonoside I | 0 | 0 | 0 | 0.22±0.01 | 0 | 0.26±0.01 |
| 05 | Isotachioside | 0.04±0.001 | 0.05±0.001 | 0 | 0 | 0 | 0 |
| 06 | Caffeic acid hexoside | 0.39±0.02 | 0.47±0.03 | 0 | 0 | 0 | 0 |
| 07 | Coumaroylquinic acid | 0.34±0.01 | 0.53±0.03 | 0 | 0 | 0 | 0 |
| 08 | Quercetin dihexoside | 0.19±0.001 | 0.18±0.01 | 0.28±0.06 | 0.39±0.02 | 0.36±0.01 | 0.47±0.02 |
| 09 | (Epi)catechin | 1.15±0.02 | 1.06±0.05 | 0.92±0.18 | 1.09±0.05 | 1.14±0.03 | 1.28±0.04 |
| 10 | Maclurin | 0.01±0.001 | 0.01±0.001 | 0.14±0.03 | 0.22±0.01 | 0.18±0.01 | 0.26±0.01 |
| 11 | Trihydroxybenzophenone O/C-hexoside | 0.2±0.01 | 0.2±0.01 | 0.17±0.04 | 0.23±0.01 | 0.22±0.01 | 0.27±0.01 |
| 12 | Procyanidin C | 0.18±0.001 | 0.17±0.01 | 0.19±0.04 | 0.24±0.02 | 0.23±0.01 | 0.27±0.01 |
| 13 | Mangiferin | 0.12±0.001 | 0.17±0.05 | 0.23±0.07 | 0.47±0.02 | 0.33±0.08 | 0.52±0.01 |
| 14 | Kaempferol hexoside | 1.39±0.01 | 1.33±0.09 | 0.94±0.2 | 0.86±0.04 | 0.94±0.04 | 0.95±0.03 |
| 15 | Procyanidin B II | 0 | 0 | 0.17±0.03 | 0.93±0.04 | 0.64±0.03 | 1.06±0.03 |
| 16 | Quercetin hexoside | 2±0.03 | 2.13±0.13 | 1.71±0.33 | 2.06±0.08 | 2.16±0.04 | 2.41±0.07 |
| 17 | Quercetin acetylhexoside | 0.91±0.01 | 0.98±0.05 | 0.69±0.13 | 0.92±0.04 | 0.91±0.02 | 1.08±0.03 |
| 18 | TetraOH,MeOxanthone hexoside | 0 | 0 | 0.25±0.05 | 0.45±0.02 | 0.34±0.01 | 0.56±0.02 |
| 19 | Neolancerin | 0.05±0.001 | 0.06±0.001 | 0.06±0.01 | 0 | 0.09±0.001 | 0 |
| 20 | DiHO,diMeOxanthone 1 | 0.14±0.001 | 0.17±0.01 | 0.21±0.04 | 0.51±0.02 | 0.32±0.01 | 0.68±0.04 |
| 21 | TtriHO,MeOxanthone malonylhexoside | 0.11±0.001 | 0.15±0.01 | 0.16±0.03 | 0.05±0.001 | 0.21±0.03 | 0.07±0.001 |
| 22 | DiOH,diMeOxanthone hexoside | 0.18±0.001 | 0.23±0.01 | 0.1±0.02 | 0 | 0.24±0.04 | 0 |
| 23 | TetraHOxanthone | 0 | 0 | 0.27±0.08 | 0.78±0.04 | 0.31±0.02 | 0.97±0.03 |
| 24 | TetraHO,MeOxanthone | 0.04±0.001 | 0.05±0.001 | 0.09±0.02 | 0.24±0.01 | 0.14±0.01 | 0.26±0.01 |
| 25 | DiMeO,Hoxanthone | 0.02±0.001 | 0.13±0.02 | 0.13±0.03 | 0.26±0.01 | 0.19±0.01 | 0.47±0.07 |
| 26 | Bisxanthone | 0 | 0 | 0.24±0.05 | 0.37±0.02 | 0.33±0.01 | 0.46±0.02 |
| 27 | Hyperxanthone B | 0 | 0 | 0.25±0.06 | 0.31±0.01 | 0.4±0.02 | 0.53±0.11 |
| 28 | TriHO,diMeOxanthone | 0.01±0.001 | 0.02±0.01 | 0.02±0.001 | 0.23±0.001 | 0.03±0.001 | 0.21±0.07 |
| 29 | TriHO,MeOxanthone 1 | 0.01±0.001 | 0.04±0.001 | 0.23±0.05 | 0.68±0.03 | 0.38±0.02 | 0.87±0.05 |
| 30 | Hyperxanthone A | 0.03±0.001 | 0.02±0.001 | 0.19±0.04 | 0.52±0.02 | 0.3±0.01 | 0.66±0.02 |
| 31 | Cadensin G | 0.16±0.001 | 0.17±0.001 | 0.48±0.09 | 1.11±0.04 | 0.76±0.03 | 1.3±0.05 |
| 32 | DiHO,diMeOxanthone 3 | 0.03±0.001 | 0.02±0.001 | 0 | 0.07±0.001 | 0 | 0.23±0.07 |
| 33 | Hyperxanthone C | 0 | 0 | 0.15±0.04 | 0.41±0.04 | 0.27±0.04 | 0.49±0.02 |
| 34 | DiHO,diMeOxanthone 4 | 0 | 0 | 0.05±0.01 | 0.15±0.01 | 0.09±0.01 | 0.19±0.01 |
| 35 | Gemixanthone A | 0 | 0 | 0.13±0.03 | 0.31±0.01 | 0.22±0.05 | 0.42±0.03 |
| 36 | Toxyloxanthone B | 0 | 0 | 0.18±0.04 | 0.71±0.02 | 0.33±0.01 | 0.92±0.06 |
| 37 | Hyperxanthone E | 0 | 0 | 0.32±0.08 | 1.07±0.04 | 0.51±0.02 | 1.45±0.15 |
| 38 | Gancaonin O | 0 | 0 | 0.05±0.01 | 0.15±0.01 | 0.09±0.01 | 0.19±0.01 |
| 39 | 2-deprenyl-7-HO-rheediaxanthone | 0.04±0.01 | 0.02±0.001 | 0.82±0.15 | 2.83±0.09 | 1.52±0.05 | 4.32±0.67 |
| 40 | Hyperxanthone D | 0 | 0 | 0.24±0.1 | 0.42±0.02 | 0.21±0.01 | 0.53±0.01 |
| 41 | Paxanthone | 0.05±0.001 | 0.04±0.001 | 0.32±0.08 | 1.29±0.04 | 0.5±0.03 | 1.57±0.06 |
| 42 | TriHO,MeOprenylxanthone 4 | 0.2±0.001 | 0.19±0.001 | 0.34±0.05 | 0.87±0.04 | 0.5±0.02 | 1.02±0.05 |
| 43 | 6-deoxyisojacareubin | 0 | 0 | 0 | 0.36±0.02 | 0 | 0.51±0.03 |
| 44 | DiHO,diMeOprenylxanthone | 0 | 0 | 0 | 0.39±0.02 | 0 | 0.54±0.03 |
| 45 | Isojacareubin | 0.01±0.001 | 0.01±0.001 | 0.08±0.02 | 0.49±0.08 | 0.22±0.04 | 0.65±0.08 |
| 46 | Gamma-Mangostin | 0.01±0.001 | 0.01±0.001 | 0.07±0.02 | 0.27±0.1 | 0.18±0.02 | 0.43±0.04 |
| 47 | Garcinone B | 0.01±0.001 | 0.01±0.001 | 0.03±0.01 | 0.3±0.04 | 0.14±0.02 | 0.35±0.03 |
| 48 | Roeperanone | 0 | 0 | 0 | 0.42±0.04 | 0 | 0.61±0.04 |
